# MedNeXt for accurate medical image classification and segmentation: A lightweight transformer-style convolutional neural network

**DOI:** 10.1371/journal.pone.0340108

**Published:** 2026-01-05

**Authors:** Ziqing Xue, Pengpeng Pi, Ziyi Liu, Zhaomu Zeng, Zhiwei Sun

**Affiliations:** 1 School of Clinical Medicine, Hebei University, Baoding, Hebei, P.R. China; 2 Affiliated Hospital of Hebei University, Baoding, Hebei, P.R. China; 3 School of Intelligence Science and Technology, University of Science and Technology Beijing, Beijing, P.R. China; 4 Department of Neurosurgery, Jiangxi Provincial People’s Hospital, The First Affiliated Hospital of Nanchang Medical College, Nanchang, Jiangxi, P.R. China; 5 Department of Toxicology and Sanitary Chemistry, School of Public Health, Capital Medical University, Beijing, P.R. China; Khalifa University, UNITED ARAB EMIRATES

## Abstract

Transformer-based deep learning architectures have achieved notable success across various medical image analysis tasks, driven by the global modeling capabilities of the self-attention mechanism. However, Transformer-based methods exhibit significant computational complexity and a large number of parameters, rendering them challenging to apply effectively in practical medical scenarios. Compared with Transformers, large-kernel Convolutional Neural Networks (CNNs) and Multi-Layer Perceptrons (MLPs) offer more efficient inference while retaining global contextual awareness. Therefore, we rethink the role of large-kernel CNNs and MLPs in medical image analysis and leverage them to replace the heavy self-attention operation, to strike a better balance between performance and efficiency. Specifically, we propose backbone models for medical image classification and segmentation, featured by three lightweight modules: Linear Attention Feed Forward Network (FFN) for enhancing lesion features, Spatial Encoding Module for integrating multi-scale lesion information, and Smooth Depth-Wise Convolution (DwConv) FFN for efficient interaction of channel features. Composed solely of lightweight convolutional and MLP operations, our method achieves a better balance between performance and efficiency, validated by the superior performances on five datasets with varying data scales and diseases, with 98.39% on SARS-COV2-CT-Scan, 98.12% on Monkeypox Skin Lesion Dataset, 98.58% on Large COVID-19-CT scan slice, 79.45% on Synapse and 91.28% on ACDC. The low computational cost, high-performance with limited training data, and generalizability to various of medical tasks make the proposed method a promising and practical solution for medical image classification and segmentation.

## Introduction

Medical imaging serves as a critical tool for clinical screening, diagnosis, treatment guidance, and evaluation. Conventional manual imaging diagnosis heavily relies on clinicians’ experience, which is also labor-intensive. To alleviate this burden, automatic medical image classification and segmentation are in high demand [1]. Deep learning methods have achieved remarkable results for a variety of tasks related to medical imaging [2,3]. Recently, Transformer-based structures rapidly emerge as a hot spot of research, which have demonstrated impressive performance in various computer vision tasks with its superior global context-awareness capabilities [4–6].

Previous methods mainly focuses on improving Transformers for general computer vision tasks, such as introducing pyramid structures [6], exploiting the inductive bias of CNNs [7], and reducing computational complexity [8]. However, for medical image analysis, existing methods still suffer from the following problems: (1) Heavy dependence on data: Most recent works have been conducted for large datasets, various variants of Transformers, MLPs and large-kernel CNNs (e.g., Swin-Transformer-v2 [9], ResMLP [10], ConvNeXt [11]) show severe performance degeneration on small datasets. (2) High computational complexity and redundant parameters: In medical image segmentation tasks, previous works [12–14] compensate for the lack of long-range modeling of convolutions by using the Transformers as encoders. However, the computational complexity of self-attention is , which will be extremely slow when dealing with high-dimensional data like medical images [15]. (3) Lack of multi-scale information within the same stage: Previous works [16,17] have relied on a pyramid structure for the design of the encoder, which only considered multi-scale information across different stages or layers, while ignored multi-scale information within the same stage or layer. It is crucial for semantic segmentation, since a dense prediction task relies more on multi-scale information and rich semantic features to capture objects of different sizes in an image.

Therefore, we believe a good visual model for medical image analysis should have the following characteristics: (1) Handling datasets with various scales; (2) Balancing performance and efficiency; (3) Capturing multi-scale information from both the same and different stages. To meet these requirements, we propose a lightweight transformer-style convolutional neural network named MedNeXt , which leverages large-kernel Convolutional Neural Networks (CNNs) and Multi-Layer Perceptrons (MLPs) to replace the heavy self-attention operation for a better balance between performance and efficiency. Three novel modules are proposed: Linear Attention Feed Forward Network (LAFFN) for enhancing lesion features, Spatial Encoding Module (SEM) for integrating multi-scale lesion information, and Smooth Depth-Wise Convolution FFN (SDFFN) for efficient interaction of channel features.

Specifically, to fully consider the importance of local modeling ability versus context-global modeling ability for visual models, we first constructed the feature enhancement module LAFFN by mimicking the paradigm of linear Transformer through parallel MLP branches. LAFFN learns long-distance information interaction through a dynamic modeling approach similar to Transformer with only computational complexity. For the SEM, multi-scale information is aggregated in asymmetric depth-wise convolutions with different kernel sizes and MLPs. Compared with classical CNNs which usually adopt a static modeling manner, the proposed SEM can adaptively process the input information through a dynamic modeling pipeline similar to Transformer without relying on the heavy self-attention. SDFFN is constructed for efficient channel-mixing with smooth dimensional transition, which offers superior performance compared to the original FFN, while significantly reducing the number of parameters.

Benefiting from the three proposed modules, our MedNeXt can handle datasets with various scales and achieve a better balance between performance and efficiency. With only 59% of the computational cost of Swin-Transformer v2 (1.90G vs. 3.24G), MedNeXt was able to improve the accuracy on the SARS-COV2 Ct-Scan, Monkeypox Skin Lesion, and Large COVID-19 CT scan slice medical image classification datasets by 3.83%, 5.01%, and 1.39%, respectively. And MedUNeXt, an efficient medical image segmentation network based on MedNeXt, still performs well on Synapse and ACDC, two mainstream medical image segmentation datasets. Thanks to the inductive bias of convolutions and the global context modeling ability of MLPs, our MedNeXt has excellent performance on different kinds of datasets without suffering from the issue that CNN is only suitable for small data sets and Transformer under-performs on small data sets due to the lack of inductive bias. Our contributions are summarized in the following three aspects:

- Three new modules are proposed: the Linear Attention FFN (LAFFN) for enhancing lesion features, the Spatial Encoding Module (SEM) for integrating multi-scale lesion information, and the Smooth DwConv FFN (SDFFN) for efficient interaction of channel features.
- The medical image classification network MedNeXt and the segmentation network MedUNeXt are proposed. MedNeXt and MedUNeXt only rely on inexpensive convolution and MLP operations, enabling more efficient inference. In addition, MedNeXt and MedUNeXt consider multi-scale information both across different stages, and within the same stage.
- MedNeXt achieves the best comprehensive performance on the three medical image classification datasets of SARS-COV2 Ct-Scan, Monkeypox Skin Lesion dataset, and Large COVID-19 CT scan slice. MedUNeXt showed superior performance on Synapse and ACDC. MedNeXt and MedUNeXt strike a better balance between performance and efficiency than previous works.

## Related works

Deep learning-based computer vision methods have made significant advancements in the field of medical image analysis. In this section, we begin with a brief overview of the basic principles of classification and segmentation with related literature. Afterwards, methods in medical image classification and segmentation are reviewed, respectively.

### Classification and segmentation.

Image classification is a fundamental task in computer vision and has been widely applied across domains such as biometrics, geoscience and remote sensing, disaster monitoring, medical diagnosis, and agricultural automation [18,19]. It is the task of assigning an input image to one category from a predefined set of labels.

The emerging of deep learning-based approaches has significantly improved classification accuracy with Convolutional Neural Networks (CNNs) long dominating this field. CNNs, exemplified by AlexNet [20], pioneered the application of deep learning in computer vision. Subsequent models [21–24] enhance network design by expanding width or increasing depth. Vision Transformer (ViT) further improved traditional CNNs by introducing self-attention mechanisms [4]. Exemplified by the ViT, various subsequent models [9,25,26] are proposed. DeiT [25] is a transformer-based model that uses a combination of encoder and decoder layers to process image patches. CaiT [26] leverages cross-attention to combine information from different patches of the input image. Swin-Transformer-v2 [9] divides the input image into non-overlapping patches, and then process them in a hierarchical manner by multiple transformer layers.

In contrast to image classification, which assigns a single image-level label to an entire image, image segmentation assigns a semantic label to every pixel, producing a dense pixel-wise segmentation map over a predefined set of categories [27]. Pioneer deep-learning-based method [28] is limited by fully connected layers, which deplete the spatial information. Fully Convolutional Networks (FCNs) is proposed to address this limitation [29]. Transformer-based methods are also extended to the segmentation task [30], incorporating pyramid structure [31,32], dual branches [33] and semantic context encoder [34], to capture the long-range semantic dependencies.

### Medical image classification.

Diagnosing disease from medical images (e.g., X-ray, CT, and MRI) can be formulated as a classification task. Medical image classification focuses on learning domain-specific knowledge to aid clinical decision-making. Therefore, deep models are typically pre-trained on large natural image datasets and fine-tuned on medical datasets for improved performance. Khan et al. proposed the CoroNet model, utilizing the pre-trained Xception architecture for COVID-19 diagnosis [35], while Yan et al. developed a hybrid CNN-RNN model that surpassed existing methods in breast cancer diagnosis [36]. These studies demonstrate the effectiveness of CNNs in local feature extraction and medical image classification.

However, CNNs have limitations due to their focus on local convolutions, lacking global context awareness. To address this, ViT extracted global features from images using self-attention mechanisms [4], but it lacks the inductive bias inherent in CNNs, making it challenging to apply directly to medical tasks. To overcome these challenges, researchers have proposed hybrid models combining CNNs and Transformers. The CNN-Swin-Transformer hybrid model proposed by Hsu et al. [37] and the TransEye model developed by Yang et al. [38] have made significant progress in improving medical image classification accuracy but still face high computational costs and efficiency issues.

In contrast, our novel approach achieves global modeling capabilities comparable to Transformers using only inexpensive convolution and MLP operations. This method maintains robust global modeling ability while significantly reducing computational costs, achieving a good balance between performance and efficiency, thereby enhancing the practical applicability of medical image classification.

### Medical image segmentation.

Distinguishing organ or lesion pixels based on the given medical images can be formulated as the medical image segmentation task [39]. It is a cornerstone of medical image analysis, enabling precise localization of lesion areas and providing essential guidance for preoperative planning. Similar to medical image classification, CNNs have long dominated the field. U-Net [40] and its variants [41,42] have been widely adopted due to their effectiveness in dissecting complex structures. These models rely on convolution operations to extract hierarchical features. However, as seen in classification tasks, the inherently local nature of convolution limits their global modeling capability. When confronted with images that require a comprehensive understanding across multiple scales for accurate segmentation, this limitation becomes a significant bottleneck.

To address this challenge, Transformer-based architectures have been introduced into medical image segmentation tasks. TransUNet [12] exemplifies a successful fusion, combining the strengths of U-Net and Transformers. By leveraging the self-attention mechanism, it captures long-range dependencies and global context, significantly enhancing segmentation accuracy. Other research has also explored various approaches to integrating Transformers in segmentation. Feng et al. [16] introduced spatial pyramid and attention mechanisms to extract targets from high-similarity backgrounds, while Cao et al. [13] investigated the feasibility of a pure Transformer model. These studies highlight the potential of Transformers to meet the global context requirements of medical image segmentation.

The above methods leverage the strengths of convolution and self-attention mechanisms, utilizing multi-scale information to enhance global modeling capability and segmentation accuracy, thereby advancing medical image segmentation. However, these methods primarily focus on multi-scale information across different stages or layers, neglecting the multi-scale information within the same stage or layer. For semantic segmentation, as dense prediction tasks rely on rich multi-scale information and semantic features to accurately capture targets of varying scales.

## Methodology

In the first subsection, we first elaborated on the overall architectural design of the proposed MedNeXt, which consists of a sequence of MED blocks. The three main components of a MED block are Linear Attention FFN for feature enhancing lesion features, Spatial Encoding Module for integrating multi-scale lesion information, and Smooth DwConv FFN for efficient interaction of channel features, as described in the following three subsections, respectively. In the last subsection, we further propose a MedUNeXt for the medical image segmentation task, which is extended from the proposed MedNeXt by adopting a U-shaped network structure.

### Overall architecture.

**Fig 1:**
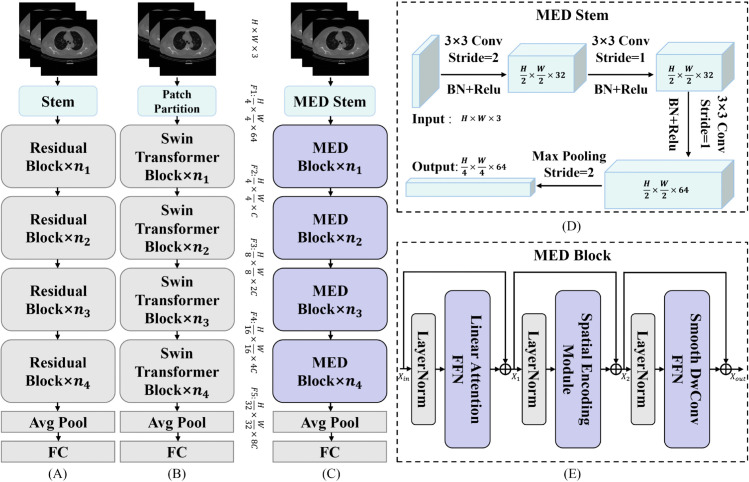
Overview of MedNeXt framework. (A) Architecture of ResNet50. (B) Architecture of Swin-Transformer. (C) Architecture of MedNeXt. (D) Detailed structure of the proposed MED Stem. (E) Detailed structure of the proposed MED Block.

The overall architecture of MedNeXt is illustrated in Fig 1. Following ResNet and Swin-Transformer as shown in Fig 1A and 1B, our MedNeXt in Fig 1C adopts a pyramid structure design. This design choice is particularly critical in medical image processing, as it enables hierarchical feature extraction, capturing the complexity and multi-scale characteristics of both anatomical structures and pathological changes. We avoid the approach of dividing medical images into smaller patches, as this could compromise the integrity of anatomical or pathological details. Instead, we directly input the entire medical image into the network. This holistic approach ensures that the model can fully leverage the inherent spatial and contextual information present in the image. Subsequently, medical images, such as computed tomography (CT), magnetic resonance imaging (MRI), or X-rays, are fed into our MED stem (as shown in Fig 1D, yielding the initial feature map ${𝐅}_{1}\in {\mathbb{R}}^{\frac{H}{4}\times \frac{W}{4}\times 64}$. Afterwards, the model is divided into four stages by downsampling operation, and *n*_*i*_ denotes the number of stacked MED blocks of the *i*-th stage. Consequently, four consecutive feature maps with different scales are obtained, noted as ${𝐅}_{2}\in {\mathbb{R}}^{\frac{H}{4}\times \frac{W}{4}\times C}$, ${𝐅}_{3}\in {\mathbb{R}}^{\frac{H}{8}\times \frac{W}{8}\times 2C}$, ${𝐅}_{4}\in {\mathbb{R}}^{\frac{H}{16}\times \frac{W}{16}\times 4C}$, and ${𝐅}_{5}\in {\mathbb{R}}^{\frac{H}{32}\times \frac{W}{32}\times 8C}$. MED stem consists of three consecutive convolutional blocks and a maximum pooling layer with stride set as 2. A convolutional block is a combination of a 3×3 convolution layer, ReLU activation function, and a batch normalization layer. The first convolution stride is set as 2 for downsampling.

The blocks in most previous Transformer-like models were designed with a two-stage structure of token-mixing and channel-mixing. The multi-head self-attention and its variants are usually used as spatial information interaction modules in the token-mixing phase, while Feed Forward networks (FFNs) are usually employed in the channel-mixing phase. Our motivation is to construct a visual model with a Transformer-like structure only relying on convolutions and MLPs. Unlike previous Transformer-like models, our MED blocks in MedNeXt are designed with a three-stage structure. Each MED block contains a Linear Attention FFN (LAFFN) for enhancing lesion features, a Spatial Encoding Module (SEM) for integrating multi-scale lesion information, and a Smooth DWConv FFN (SDFFN) for efficient interaction of channel features. We will elaborate on these model components in the following sections.

As shown in Fig 1E, the workflow of the MED block can be formulated as follows:

$${𝐗}_{1}={𝐗}_{\text{in}}+LAFFN(LayerNorm({𝐗}_{\text{in}}))$$ (1)

$${𝐗}_{2}={𝐗}_{1}+SEM(LayerNorm({𝐗}_{1}))$$ (2)

$${𝐗}_{\text{out}}={𝐗}_{2}+SDFFN(LayerNorm({𝐗}_{2}))$$ (3)

where ${𝐗}_{\text{in}}$ and ${𝐗}_{\text{out}}$ represent the input and output of each MED block, respectively. ${𝐗}_{1}$ and ${𝐗}_{2}$ are intermediate transmission values. Layer normalization [43] is used to normalize data when each component carries out information interaction.

### Linear attention feed forward network.

**Fig 2:**
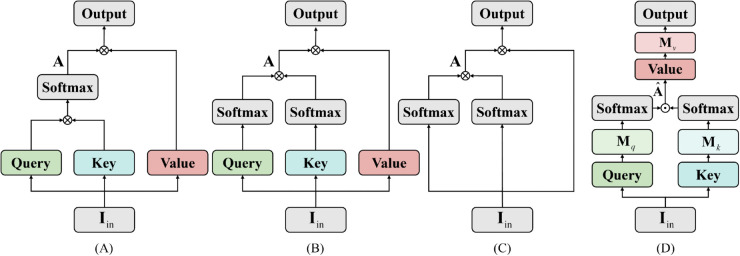
Overview of linear attention FFN (⊗: Matrix multiplication; ⊙: Hadamard product). (A) Self-attention. (B) Linear-attention. (C) Simplified linear-attention. (D) Proposed Linear Attention Feed Forward Network (LAFFN).

In medical image analysis, precise and efficient feature extraction is crucial. Medical images (such as CT scans, MRIs, and X-ray images) often contain complex anatomical structures and subtle pathological features. To effectively process these complex data, this subsection introduces the proposed Linear Attention Feed Forward Network (LAFFN), as shown in Fig 2. First, we analyze the commonly used self-attention mechanism. Although self-attention performs excellently in capturing global relationships, its computational complexity is relatively high, especially when applied to large-scale medical image datasets. To address this issue, we introduce the linear attention mechanism, which eliminates the softmax operation to achieve linear complexity, significantly reducing computational overhead while retaining the ability to capture global features. Subsequently, we simplify the traditional self-attention mechanism by equating the query, key, and value matrices with the input features. This simplification, which considers the specific characteristics of medical images, improves feature extraction efficiency and relevance, particularly in the identification of lesion areas and tissue boundaries. Finally, LAFFN implements this simplified self-attention paradigm through three linear layers with learnable parameters, focusing on enhancing key features in medical images.

The self-attention mechanism is the core component of the Transformer architecture. As shown in Fig 2A, let the input feature map be denoted as ${𝐈}_{\text{in}}\in {\mathbb{R}}^{N\times C}$, where *N* = *H* × *W* denotes the number of spatial elements, and *C* is the channel dimension.

In the standard self-attention formulation, the input is first linearly projected into three matrices: the query matrix $𝐐\in {\mathbb{R}}^{N\times C}$, the key matrix $𝐊\in {\mathbb{R}}^{N\times C}$, and the value matrix $𝐕\in {\mathbb{R}}^{N\times C}$. The attention weights are computed via dot-product similarity followed by softmax normalization:

$$𝐀=softmax({QK}^{T}),\;𝐀\in {\mathbb{R}}^{N\times N}.$$ (4)

The final output is obtained by applying the attention weights 𝐀 to the value matrix:

$$\text{Output}=AV,\;\text{Output}\in {\mathbb{R}}^{N\times C}.$$ (5)

However, this standard attention involves computing a similarity matrix of shape ${\mathbb{R}}^{N\times N}$, resulting in a computational complexity of $𝒪(N^{2})$, which can be prohibitive for high-resolution feature maps.

To address this, we adopt a more efficient approximation strategy, illustrated in Fig 2B, where we apply softmax normalization independently to the query and key matrices along different dimensions: the channel dimension *C* for **Q**, and the spatial dimension *N* for **K**. This yields a factorized attention formulation:

$$𝐀=softmax({𝐐}^{T}{)}^{T}\cdot softmax(𝐊{)}^{T},\;𝐀\in {\mathbb{R}}^{N\times N},$$ (6)

where softmax() operates on matrices of shape ${\mathbb{R}}^{N\times C}$, reducing the complexity to 𝒪(N) since C≪N. Furthermore, as shown in Fig 2C, we can simplify the structure further by reusing the input ${𝐈}_{\text{in}}$ for all three projections, i.e., $𝐐=𝐊=𝐕={𝐈}_{\text{in}}$, yielding:

$$𝐀=softmax({𝐈}_{\text{in}}^{T}{)}^{T}\cdot softmax({𝐈}_{\text{in}}{)}^{T},\;𝐀\in {\mathbb{R}}^{N\times N}.$$ (7)

To further improve efficiency and parameterization flexibility, we propose LAFFN, as depicted in Fig 2D, which mimics the self-attention mechanism using three learnable linear layers. Given the input ${𝐈}_{\text{in}}\in {\mathbb{R}}^{N\times C}$, we first project it into two branches via linear transformations, followed by softmax operations along the *C* and *N* dimensions, respectively. We then compute the attention-like matrix $\hat{𝐀}\in {\mathbb{R}}^{N\times C}$ via element-wise (Hadamard) product. Fig 2(D) an be formulated as follows:

$$𝐐={𝐈}_{\text{in}}{𝐌}_{q},\;𝐊={𝐈}_{\text{in}}{𝐌}_{k},$$ (8)

$$𝐕=\hat{𝐀}=softmax({𝐐}^{T}{)}^{T}⊙softmax(𝐊),$$ (9)

$$\text{Output}=𝐕{𝐌}_{v},\;\text{Output}\in {\mathbb{R}}^{N\times C},$$ (10)

where ${𝐌}_{q}$, ${𝐌}_{k}$, and ${𝐌}_{v}\in {\mathbb{R}}^{C\times C}$ are the weight matrices of three independent linear layers. And the the attention-like matrix $\hat{𝐀}$ is directly regarded as the value matrix **V**. Compared to traditional self-attention or Feed-Forward Network (FFN) modules, our approach is computationally more efficient, retains dynamic feature adaptability, and maintains linear complexity with respect to input size.

### Spatial encoding module

**Fig 3:**
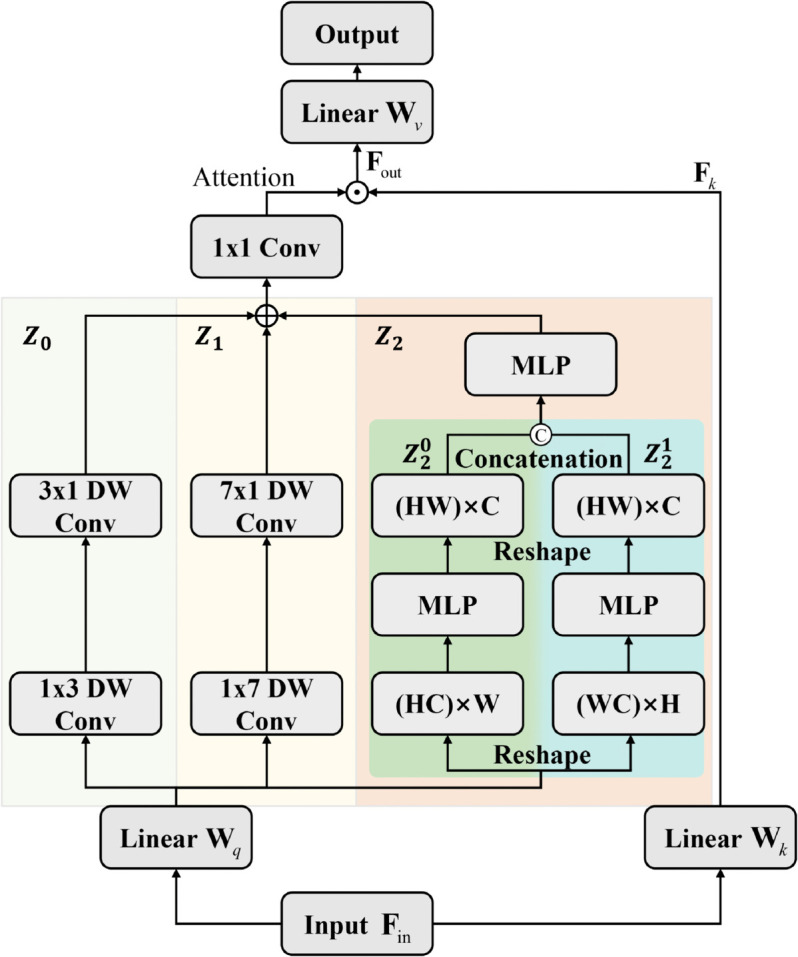
Spatial encoding module (⊙: Hadamard product).

In medical image analysis, accuracy directly impacts patient diagnosis and treatment. The structure of the proposed Spatial Encoding Module (SEM) is shown in Fig 3. Medical images, such as CT scans, MRIs, and X-rays, contain complex visual information, with significant variations in the size, shape, and location of lesions and anatomical structures. For example, in the analysis of COVID-19 or monkeypox images, small lesions may be difficult to distinguish from surrounding healthy tissue, while larger lesions present significant differences at various scales.

Unlike Transformer variants applied in non-medical domains, we recognize the unique characteristics of medical imaging, particularly the need for efficient utilization of computational resources and real-time analysis in clinical settings. Therefore, we employ low-cost convolutions combined with Multi-Layer Perceptrons (MLPs) as the encoding module for spatial information. This approach not only reduces computational complexity but also optimizes feature extraction to suit the specific characteristics of medical images.

Our SEM plays a crucial role in aggregating multi-scale information, addressing a key limitation in traditional methods that often fail to collect multi-scale details within the same stage. Medical semantic segmentation tasks rely on rich multi-scale information and robust semantic features to accurately identify and segment targets of varying sizes, from small lesions to large masses. By integrating multi-scale features, SEM provides a more comprehensive feature representation for downstream classification and segmentation tasks. Denoting the input as ${𝐅}_{\text{in}}\in {\mathbb{R}}^{H\times W\times C}$, the workflow of the SEM is formulated as follows:

$${𝐅}_{k}={𝐅}_{\text{in}}{𝐖}_{k},\;{𝐅}_{k}\in {\mathbb{R}}^{H\times W\times C}$$ (11)

$$\text{Attention}=\text{Conv}\left(\sum_{i=0}^{2}Z_{i}({𝐅}_{\text{in}}{𝐖}_{q})\right)$$ (12)

$${𝐅}_{\text{out}}=\text{Attention}⊙{𝐅}_{k}$$ (13)

$$\text{Output}={𝐅}_{\text{out}}{𝐖}_{v}$$ (14)

where ${𝐖}_{k}$, ${𝐖}_{q}$, and ${𝐖}_{v}\in {\mathbb{R}}^{C\times C}$ denote the learnable parameters of the linear layers in Fig 3. *Z*_0_, *Z*_1_, and *Z*_2_ are the three branch paths. In contrast to self-attention, which calculates attention weights by measuring the similarity of the inner product of the query and key matrices, we aggregate multi-scale information from depth-wise convolutions with different kernel sizes and MLPs on a single branch path to obtain attention weights.

To calculate the attention weight in Eq. 12 in the left branch, we divide it into three branch paths: *Z*_0_, *Z*_1_, and *Z*_2_. In *Z*_0_, we replace the 3×3 depth-wise convolution with an equivalent combination of 1×3 and 3×1 depth-wise convolutions to reduce the model’s computational cost. In *Z*_1_, we use a similar strategy by replacing the 7×7 depth-wise convolution with a combination of 1×7 and 7×1 depth-wise convolutions. While the 7×7 depth-wise convolution has a larger receptive field than the 3×3 depth-wise convolution, it is still insufficient compared to the global modeling ability of self-attention.

Similar to Transformers, MLPs also have global modeling capabilities, and their relatively concise architecture opens up the possibility of deploying such models in timely medical applications. Therefore, in the branch path *Z*_2_, we provide global modeling capability for the model through MLPs. As we know, the computational complexity of the previous model, similar to MLP-Mixer, grows quadratically with the number of input features *N*, so the computational complexity was too high and the number of parameters was redundant. In branch *Z*_2_, information is exchanged by row and column, respectively. This method can not only retain the global modeling ability of the model but also effectively avoid the disadvantage of mixed interaction of all tokens in the previous MLPs architectures.

As shown in Fig 3 that the input feature is ${𝐅}_{\text{in}}\in {\mathbb{R}}^{H\times W\times C}$, we first divide the branch path *Z*_2_ into two identical paths, $Z_{2}^{0}$ and $Z_{2}^{1}$, and then reshape the input features as $𝐅\in {\mathbb{R}}^{HC\times W}$ in the constant path $Z_{2}^{0}$ and $𝐅\in {\mathbb{R}}^{WC\times H}$ in the constant path $Z_{2}^{1}$. Finally, the output matrices with the same dimension as the input features are obtained by fusing the output on two identical paths through the FC layer. Collectively, the computational complexity of our MLPs in branching path *Z*_2_ is calculated as

$$T(Z_{2})=H^{2}WC+HW^{2}C+2HWC=𝒪(N\sqrt{N}),$$ (15)

where *N* = *HW* denotes the number of input features. Compared with conventional MLPs architectures in the token-mixing stage, which has the computational complexity of $𝒪(N^{2})$, our *Z*_2_ is more lightweight.

We obtain the attention weight by aggregating the outputs of the constant paths *Z*_0_, *Z*_1_, and *Z*_2_ through a 1×1 convolution. Afterwards, the attention weight is Hadamard product with the output of another single branch to obtain the corresponding output. Finally, we use the attention weight to the Hadamard product with the output of another single branch to get the corresponding output.

Our proposed SEM simplifies self-attention by using two parallel identity branches, which is simpler and more efficient than self-attention and has lower computational complexity. In contrast to previous work, which only focused on multi-scale information across different stages or layers. While the overall structure of MedNeXt maintains the pyramid structure design, its core component SEM performs multi-scale information aggregation by depth-wise convolutions with different kernel sizes and MLPs to obtain the final output weights.

Finally, MedNeXt takes into account not only multi-scale information in different stages but also multi-scale information in the same stage, enabling it to capture rich features in objects of different sizes. In addition, convolutions have inductive bias compared to MLPs and Transformers, which gives them a natural advantage in small data sets. Because SEM aggregates information by convolutions with different sizes and MLPs, MedNeXt has excellent global and local modeling capabilities at the same layer, allowing seamless switching between small and medium or large datasets without the previous situation where the model was only applicable for single-scale datasets.

### Smooth DwConv feed forward network

**Fig 4:**
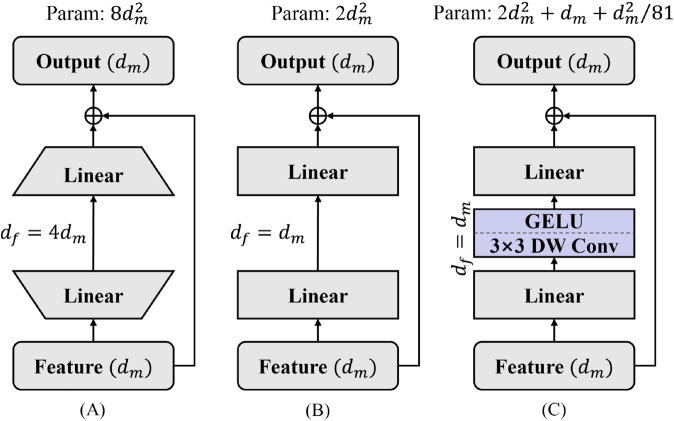
Smooth DwConv feed forward network. (A) Traditional FFN design. (B) Smooth FFN. (C) Proposed Smooth DwConv-Feed Forward Network (SDFFN).

Similar to the structure of Transformer-like models, we use a feed-forward network (FFN) to make nonlinear changes to the information space of attention aggregation so as to improve the overall representation ability of the model. The traditional FFN design is shown in Fig 4(a), an inverse bottleneck structure that raises dimensions and then reduces dimensions, and its expansion coefficient is usually 4. We assume that the input feature dimension is *d*_*m*_, so the number of traditional FFN parameters is $8d_{m}^{2}$. As the feature map of the model continues to shrink and the feature dimension continues to increase, the number of parameters of FFN will continue to rise with the feature dimension. Considering the expansion of the model in timely medical applications, and in order to better balance the efficiency and performance of the model, we did not adopt the traditional FFN design but proposed the improved FFN design Smooth DwConv-Feed Forward Network (SDFFN).

In SDFFN, instead of extending the hidden dimensions of FFN, we use a smooth transition. To increase the model’s overall expressiveness, we added 3 × 3 depth-wise convolution between the two MLP layers. The overall number of parameters in SDFFN can be approximately equivalent to: $2d_{m}^{2}+d_{m}+\frac{d_{m}^{2}}{81}$. If the dimension of the feature map is 512, the number of parameters of traditional FFN is 2,097,152, while that of SDFFN is 528,036. Follow-up experiments will prove that SDFFN performs better than traditional FFN while significantly reducing the number of parameters.

### MedUNeXt

**Fig 5:**
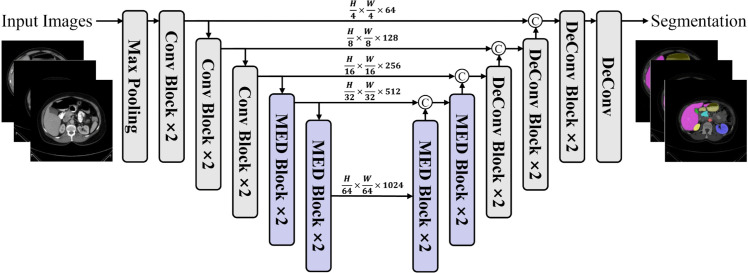
Overview of MedUNeXt framework.

The architecture of MedUNeXt is illustrated in Fig 5. Similar to traditional UNet variants, we adopt the classic encoder-decoder architecture. To address the unique challenges presented by medical imaging, we leverage the proposed MED block. In contrast to architectures that emphasize the global modeling capability of Transformers, our design prioritizes computational efficiency and the preservation of fine-grained spatial details—critical elements for medical image analysis. The MED block is deployed in deeper stages, playing a crucial role in global context modeling. This module enhances global context modeling by aggregating multi-scale features, a capability essential for handling significant size variations of lesions and anatomical structures. For example, in ACDC cardiac imaging, small structures require fine-grained scales for accurate segmentation, while larger organs or lesions require coarser scales for proper localization. The MED module achieves this balance by producing feature weights through convolutions with different kernel sizes and MLPs within the same network stage.

In the encoder stage of MedUNeXt, input feature maps are initially downsampled using a max pooling layer, followed by three consecutive convolution blocks and two consecutive MED blocks. Each convolution block comprises two convolutional sub-blocks, each containing a 3 × 3 convolution and batch normalization layer and a ReLU activation function. At the end of each convolution block and MED block, we down-sample through a 3 × 3 convolution with a stride of 2. The decoder stage mirrors the encoder structure and we perform feature map upsampling by de-convolution. Additionally, skip connections are utilized to fuse encoder and decoder features, mitigating the loss of spatial information.

Compared to previous hybrid models based on convolutions and Transformers or pure Transformers models. Our MedUNeXt is constructed by pure convolutions and MLPs, which is more unified, efficient, and convenient while having multi-scale context awareness ability from local to global.

## Experiments and results

### Dataset

To validate the effectiveness of the proposed method, experiments are carried on five datasets, including three datasets of image classification (i.e., SARS-COV2 CT-Scan [44], Monkeypox Skin Lesion Dataset [45] and Large COVID-19 CT scan slice [46]) and two datasets of image segmentation (i.e., Synapse [47] and ACDC [48]). The details of these datasets are summarized in Table 1. Besides, comparisons with mainstream methods are performed to validate the superiority of our method. Finally, to validate the effectiveness of each proposed component, detailed ablation studies are presented experiments on the SARS-COV2 CT-Scan and ACDC.

**Table 1:** Descriptions and split settings of five used datasets.

Dataset	Class	Training set	Validation set	Test set	Total
SARS-COV2-CT-Scan	Normal	737 images	246 images	246 images	1229 images
	COVID-19	751 images	251 images	250 images	1252 images
Monkeypox Skin Lesion Dataset	Monkeypox	999 images	215 images	214 images	1428 images
	Other	1234 images	265 images	265 images	1764 images
Large COVID-19-CT scan slice	Normal	4136 images	1379 images	1378 images	6893 images
	COVID-19	4556 images	1519 images	1518 images	7593 images
Synapse	-	18 cases	-	12 cases	30 cases
ACDC	-	70 cases	10 cases	20 cases	100 cases

**SARS-COV2 Ct-Scan**. The dataset consists of 1252 CT lung images from COVID-19 positive patients and 1230 CT lung images from COVID-19 negative subjects, collected from the Hospital of São Paulo, Brazil. All images were resized to 224 × 224 and augmented with random cropping and horizontal flipping. The dataset has balanced sample distribution and moderate size, making it suitable for evaluating method effectiveness in single-disease classification, particularly for COVID-19 diagnosis.

**Monkeypox Skin Lesion Dataset**. It is a publicly available collection of skin images that were originally collected by Shams Nafisa Ali and colleagues to aid in the diagnosis of monkeypox. The dataset has since been expanded through data enhancement techniques to include a total of 3192 skin images, with 1428 images from patients with monkeypox and 1764 images from patients with other skin conditions. All images have been resized to a uniform size of 224 × 224. The dataset contains images of monkeypox and other skin diseases, with an expanded and balanced sample size, making it suitable for evaluating the method’s generalization capability in skin disease classification, particularly for identifying subtle differences.

**Large COVID-19 CT scan slice**. The Large COVID-19 CT scan slice dataset is a collection of CT scan images used for the diagnosis of COVID-19. The dataset is compiled by Maftouni et al. and includes 7593 COVID-19 images and 6893 normal images from 466 positive patients and 604 negative patients. The resize and split settings of this dataset are the same as those of the above datasets. The dataset contains a large number of COVID-19 and normal CT images from different patients, making it suitable for testing the method’s applicability to large-scale datasets and its efficiency in handling extensive data.

**Synapse**. The Synapse dataset is a multi-organ segmentation dataset used in the MICCAI 2015 open challenge. This dataset consists of 30 clinically acquired, contrast-enhanced abdominal CT cases. In line with the protocol established in [47], 18 cases are allocated for training and 12 for testing. Each image in the dataset is annotated to include 8 key abdominal organs: the aorta, gallbladder, spleen, left kidney, right kidney, liver, pancreas, and stomach. To assess the performance of our method on this dataset, we employ the Dice Similarity Coefficient (DSC) and the 95% Hausdorff Distance (HD95) as evaluation metrics. The dataset includes annotations for 8 abdominal organs, making it suitable for evaluating the method’s global and local modeling capabilities in multi-organ segmentation of complex structures, particularly for targets of varying scales.

**ACDC**. The ACDC dataset is a publicly available dataset for heart segmentation, consisting of 100 samples. The dataset includes three segmentation objects: the myocardium (MYO), left ventricle (LV), and right ventricle (RV). Following the convention, we also use the Dice similarity coefficient (DSC) to evaluate various methods. This dataset focuses on heart segmentation, including annotations for the left ventricle, right ventricle, and myocardium, making it suitable for evaluating the method’s accuracy and boundary recognition capabilities in heart region segmentation.

### Experimental settings

**Medical Image Classification**: To evaluate the image classification performance, the precision, recall, F1, Accuracy and AUC values, based on the true positive (TP), true negative (TN), false positive (FP), and false negative (FN) values, are present. Specifically, these metrics are defined as follows:

$$\text{Precision}=\frac{TP}{TP+FP}\times 100\%$$ (16)

$$\text{Recall}=\frac{TP}{TP+FN}\times 100\%$$ (17)

$$F1=\frac{2TP}{FP+2TP+FN}\times 100\%$$ (18)

$$\text{Accuracy}=\frac{TP+TN}{TP+TN+FP+FN}\times 100\%$$ (19)

In addition, AUC (Area under Curve) refers to the area under the ROC (Receiver Operating Characteristic) curve, with values ranging between 0.5 and 1. Among them, F1, Accuracy and AUC are comprehensive metrics measuring the classification performance. For the implementation details, the proposed MedNeXt is optimized using AdamW [49], and the initial learning rate and initial momentum is set to $6\times 10^{-4}$ and 0.9, respectively. Additionally, the learning rate is adjusted via a cosine scheduler and the weight decay factor is set as 0.05, with a total training period of 300 epochs.

**Medical Image Segmentation**: Following the convention [12], we evaluated the performance of the corresponding methods using the Dice similarity coefficient (DSC) and the 95% Hausdorff distance (HD95). DSC is used to evaluate the overlap between the predicted segmentation and the ground truth labels, while HD95 is used to assess the similarity between the predicted and true segmentation boundaries. A higher DSC value indicates a greater overlap between the prediction and the ground truth, leading to better segmentation performance. Conversely, a lower HD95 value signifies that the predicted segmentation is closer to the true boundary, indicating superior segmentation quality.

$$\text{DSC}=\frac{2\times |A\cap B|}{\left|A|+|B\right|}\times 100\%$$ (20)

$$HD_{95}(A,B)=\max({\text{percentile}}_{95}(d(A,B)),{\text{percentile}}_{95}(d(B,A)))$$ (21)

In the segmentation experiments, we optimized the model using Adam [50] and set the initial learning rate to $1\times 10^{-4}$, batch size to 12, and total training epochs to 100. All code was written in Python 3.6 and PyTorch 1.8.1.

### Classification task: Research on SARS-COV2 Ct-scan

As show in Table 2, MedNeXt has demonstrated significant advantages in both medical diagnostic performance and resource efficiency for COVID-19 patients, particularly in the application of the SARS-CoV-2 CT scan dataset, highlighting its unique value in COVID-19 diagnosis. In terms of medical diagnostic performance, MedNeXt achieves a Precision of 100%, meaning that all cases predicted as COVID-19 positive are true positives (True Positives, TP), completely eliminating false positives (False Positives, FP = 0). In the context of COVID-19 diagnosis, such a high Precision is crucial as it effectively prevents healthy individuals from being wrongly isolated or receiving unnecessary treatments due to misdiagnosis, thereby reducing medical resource waste and minimizing the psychological burden on patients. Furthermore, MedNeXt’s Recall reaches 96.75%, indicating a high detection rate for actual positive cases, with only 3.25% of cases being missed (False Negatives, FN). Given the high transmissibility of COVID-19, this low false negative rate significantly reduces the risk of potential virus spread, which is especially critical in the early stages of epidemic control. In addition, MedNeXt’s overall performance is further reflected in its F1-score (98.35%) and AUC (0.9988), indicating that the model excels in balancing false positives and false negatives, achieving industry-leading performance, particularly in detecting suspected cases while maintaining both high sensitivity and high specificity.

**Table 2:** Classification task: Comparison with SOTA methods on SARS-COV2-CT-scan.

Method	Params (M)↓	FLOPs (G)↓	Precision↑	Recall↑	F1↑	Accuracy↑	AUC↑
ResNet50 [24]	25.56	4.11	97.51%	95.53%	96.51%	96.57%	0.9958
ResNet18 [24]	11.69	1.82	95.93%	**97.97%**	97.97%	97.98%	0.9969
DenseNet121 [51]	7.97	2.87	96.31%	95.68%	97.86%	98.01%	0.9987
ResMLP [10]	15.01	2.89	93.85%	93.09%	93.47%	93.65%	0.9757
MLP-Mixer [52]	29.80	6.90	93.07%	91.42%	92.23%	91.28%	0.9839
SparseMLP [53]	38.33	8.14	93.56%	94.29%	93.52%	93.26%	0.9724
ViT [4]	32.38	6.28	92.36%	92.54%	92.48%	92.84%	0.9812
Swin-Transformer-v2 [9]	21.85	3.24	99.55%	89.43%	94.06%	94.56%	0.9927
ConvNeXt-T [11]	28.60	4.50	97.37%	75.20%	84.86%	86.70%	0.9811
Patch-based CNN [54]	–	–	83.40%	86.10%	84.63%	88.90%	0.9630
**MedNeXt (Ours)**	**10.72**	**1.90**	**100%**	**96.75%**	**98.35%**	**98.39%**	**0.9988**

Bold indicates the best performance in each column. FLOPs measured in billions (G), Params in millions (M).

Moreover, the high diagnostic efficiency of MedNeXt for COVID-19 is accompanied by exceptional resource efficiency. With only 10.72M parameters and a computational cost of 1.90G FLOPs, MedNeXt outperforms many computationally intensive models (e.g., Swin-Transformer v2 with 3.24G FLOPs and ConvNeXt-T with 4.50G FLOPs), while achieving improvements of 3.83% and 11.69% in Accuracy, respectively, reaching 98.39%. This demonstrates that MedNeXt shows superior adaptability on limited computational resources and small-scale datasets compared to other complex architectures. Such an advantage is particularly important in practical COVID-19 diagnostic scenarios, such as small medical institutions with limited resources, telemedicine systems, or portable diagnostic devices, where MedNeXt can deliver rapid and accurate diagnostic results. Compared to lightweight CNN models (e.g., ResNet-18), MedNeXt achieves a Precision of 100%, an Accuracy of 98.39%, and an AUC of 0.9988, while maintaining similar computational costs, further proving its leading position in COVID-19 diagnosis.

### Classification task: Research on Monkeypox skin lesion dataset

As shown in Table 3, MedNeXt demonstrates significant advantages in the medical diagnosis of monkeypox patients. Especially when analyzing the characteristics of monkeypox - related skin lesions, its unique value in skin lesion classification and diagnosis becomes prominent. In terms of diagnostic performance, MedNeXt achieves a precision rate of 98.85%. Almost all cases determined as monkeypox - positive are true positives (TP), which significantly reduces the false positive rate (FP). In monkeypox detection, a high precision rate is of crucial importance. Misdiagnosis can lead to uninfected individuals receiving unnecessary treatment or isolation, increasing the burden on medical resources and causing psychological stress to patients. Meanwhile, the recall rate of MedNeXt is 97.74%, indicating a high detection efficiency for actual positive cases. Only 2.26% of cases are misjudged as negative (FN). Given the contagiousness of monkeypox and its public health risks, this low false negative rate can effectively reduce the risk of virus transmission by undiagnosed patients, which is of great significance for rapid detection and early - stage epidemic prevention and control.

**Table 3:** Classification task: Comparison with SOTA methods on Monkeypox skin lesion dataset.

Method	Params (M)↓	FLOPs (G)↓	Precision↑	Recall↑	F1↑	Accuracy↑	AUC↑
ResNet50 [24]	25.56	4.11	96.65%	98.11%	97.38%	97.08%	0.9927
ResNet18 [24]	11.69	1.82	97.40%	**98.87%**	98.13%	96.90%	0.9968
DenseNet121 [51]	7.97	2.87	96.59%	96.23%	96.41%	96.03%	0.9938
ResMLP [10]	15.01	2.89	93.78%	95.47%	94.57%	93.95%	0.9780
MLP-Mixer [52]	29.80	6.90	91.99%	93.31%	92.48%	91.28%	0.9734
SparseMLP [53]	38.33	8.14	92.23%	95.34%	93.35%	92.73%	0.9724
ViT [4]	32.38	6.28	91.34%	92.40%	91.86%	92.03%	0.9753
Swin-Transformer-v2 [9]	21.85	3.24	90.56%	97.04%	94.01%	93.11%	0.9841
ConvNeXt-T [11]	28.60	4.50	92.43%	95.34%	93.56%	92.89%	0.9811
Patch-based CNN [54]	–	–	90.00%	90.64%	90.31%	89.79%	0.9543
**MedNeXt (Ours)**	**10.72**	**1.90**	**98.85%**	**97.74%**	**98.29%**	**98.12%**	**0.9970**

Bold indicates the best performance in each column. FLOPs measured in billions (G), Params in millions (M).

In addition, with an F1 - score of 98.29% and an AUC of 0.9970, MedNeXt fully demonstrates its excellent ability to balance false positives and false negatives. The high F1 - score indicates its outstanding performance in both sensitivity and specificity, and the AUC value close to 1 verifies its reliability in distinguishing between positive and negative cases.

### Classification task: Research on large COVID-19 CT scan slice

MedNeXt has also demonstrated exceptional diagnostic performance and broad adaptability on the large COVID-19 CT scan dataset, providing strong evidence of its robust generalization ability on medium-sized datasets and highlighting its unique advantages in COVID-19 diagnosis. In contrast to smaller datasets such as those for SARS-CoV-2 and Monkeypox, the large COVID-19 CT scan dataset contains 14,486 images, significantly increasing the data scale and placing higher demands on the model’s ability to generalize when handling large-scale cases. As shown in Table 4, MedNeXt achieved the best performance across multiple key metrics, including Recall (99.42%), F1-score (98.80%), Accuracy (98.85%), and AUC (0.9972), while maintaining the efficient diagnostic capability observed with smaller datasets. This demonstrates MedNeXt’s ability to accurately capture COVID-19 lesion areas, ensuring diagnostic stability and reliability. Such outstanding performance is of significant clinical importance, as the high Recall value indicates a low false negative rate of only 0.58%, greatly reducing the transmission risk from undetected cases, while the high Precision and F1-score effectively balance the risks of false positives and false negatives, providing a solid technical foundation for public health interventions and early treatment.

**Table 4:** Classification task: Comparison with SOTA methods on large COVID-19-CT scan slice.

Method	Params (M)↓	FLOPs (G)↓	Precision↑	Recall↑	F1↑	Accuracy↑	AUC↑
ResNet50 [24]	25.56	4.11	95.57%	98.76%	97.14%	97.24%	0.9945
ResNet18 [24]	11.69	1.82	95.78%	96.80%	96.30%	96.46%	0.9927
DenseNet121 [51]	7.97	2.87	95.93%	97.52%	96.72%	96.77%	0.9960
ResMLP [10]	15.01	2.89	96.10%	97.83%	96.89%	96.92%	0.9956
MLP-Mixer [52]	29.80	6.90	94.57%	97.78%	96.12%	96.27%	0.9920
SparseMLP [53]	38.33	8.14	97.65%	96.62%	97.13%	97.28%	0.9968
ViT [4]	32.38	6.28	95.95%	96.32%	96.14%	96.32%	0.9941
Swin-Transformer-v2 [9]	21.85	3.24	96.87%	97.71%	97.14%	97.46%	0.9962
ConvNeXt-T [11]	28.60	4.50	**98.77%**	98.65%	98.71%	97.28%	0.9938
Patch-based CNN [54]	–	–	82.47%	90.57%	86.33%	84.17%	0.9194
**MedNeXt (Ours)**	**10.72**	**1.90**	**98.19%**	**99.42%**	**98.80%**	**98.85%**	**0.9972**

Bold indicates the best performance in each column. FLOPs measured in billions (G), Params in millions (M).

MedNeXt’s adaptability is reflected not only in its consistent performance across small and medium-sized datasets but also in its diagnostic advantages in large-scale case scenarios. Compared to mainstream modern architectures such as Swin-Transformer v2 and ConvNeXt-T, MedNeXt continues to precisely focus on COVID-19 lesion areas as the dataset size increases, while these more complex models often face difficulties with generalization or overfitting when handling smaller datasets. On the medium-sized large COVID-19 CT scan dataset, MedNeXt’s Recall increased to 99.42%, significantly outperforming Swin-Transformer v2 (97.71%) and ConvNeXt-T (98.65%), further validating its strong detection capability for positive cases. At the same time, MedNeXt’s Accuracy reached 98.85%, surpassing Swin-Transformer v2 and ConvNeXt-T by 1.39% and 1.57%, respectively, demonstrating its superior stability in COVID-19 diagnosis.

**Fig 6:**
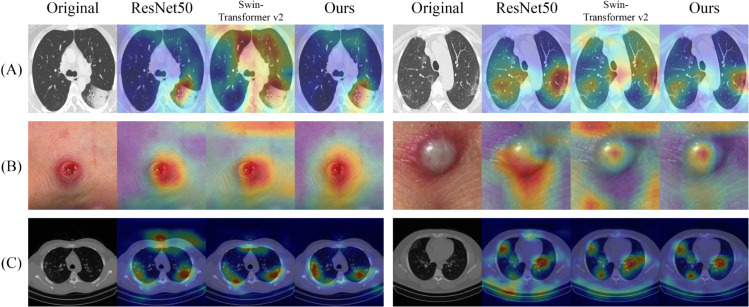
The visualization of prediction heatmaps of three medical image classification datasets. (A) SARS-COV2 Ct-scan. (B) Monkeypox skin lesion dataset. (C) Large COVID-19 CT scan slice.

In addition to the quantitative results presented in Tables 2, 3, 4, we further provide qualitative results as illustrated in Fig 6A. The prediction heatmaps are generated by ResNet50 (representing classic CNN methods), Swin-Transformer v2 (representing Transformers) and ours from left to right. For datasets with small scales, i.e., the first two rows of Fig 6B, our method and ResNet50 significantly outperform Swin-Transformer v2. Specifically, Swin-Transformer v2 may focus on area non-relevant to the diseases, while our method and ResNet50 highlight the correct regions. In contrast, for the dataset with a larger scale (the last row of Fig 6C), ResNet50 shows obvious degeneration, while our method and Swin-Transformer v2 outperform ResNet50 by highlighting the diseased regions, instead of paying attention to non-relevant regions.

**Classification Results Summary.** MedNeXt has demonstrated outstanding generalization capability and robustness across multiple experiments, proving its applicability not only to the diagnosis of different types of diseases (e.g., COVID-19 and Monkeypox) but also to datasets of varying scales (ranging from the small-scale SARS-COV2 dataset to the large-scale CT Scan dataset). As shown in Table 2-4, MedNeXt outperforms other models comprehensively in key evaluation metrics such as Precision, Recall, F1-score, Accuracy, and AUC. Moreover, leveraging its lightweight architecture, MedNeXt achieves superior diagnostic performance while operating with significantly lower computational costs compared to mainstream models. These characteristics establish MedNeXt as a versatile medical imaging analysis model capable of addressing cross-disease and cross-dataset scenarios, making it particularly suitable for resource-constrained healthcare settings and showcasing its extensive practical application potential.

### Segmentation task: Research on synapse

We compared the proposed MedUNeXt on the Synapse with previous CNN-based methods (e.g. UNet [55], AttnUNet [42], and R50UNet [12]) and Transformer-based methods (e.g., MT-UNet [14], and TransUNet [12]). As summarized in Table 5, MedUNeXt achieves an average DSC of 79.45% and an HD95 score of 23.96 mm, surpassing CNN-based methods by 1.68% to 10.64% and Transformer-based methods by 0.86% to 8.16%. These results highlight MedUNeXt’s ability to integrate the efficiency of CNNs with the global context modeling capabilities of Transformers, achieving superior performance across key segmentation metrics.

**Table 5:** Segmentation task: Comparison with SOTA methods on the synapse.

Method	DSC(%)↑	HD95(mm)↓	Aorta↑	Gallbladder↑	Kidney(L)↑	Kidney(R)↑	Liver↑	Pancreas↑	Spleen↑	Stomach↑
UNet [55]	76.85	39.70	89.07	69.72	77.77	68.60	93.43	53.98	86.67	75.58
V-Net [56]	68.81	–	75.34	51.87	77.10	**80.75**	87.84	40.50	80.56	56.98
DARR [57]	69.77	–	74.74	53.77	72.31	73.24	94.08	54.18	**89.90**	45.96
R50UNet [12]	74.68	36.87	84.18	62.84	79.19	71.29	93.35	48.23	84.41	73.92
R50AttnUNet [12]	75.57	36.97	55.92	63.91	79.20	72.71	93.56	49.37	87.19	74.95
AttnUNet [42]	77.77	36.02	89.55	**68.88**	77.98	71.11	93.57	58.04	87.30	75.75
nnUNet [58]	79.36	24.74	87.96	62.57	78.92	75.36	92.96	**66.36**	88.12	**82.60**
MT-UNet [14]	78.59	26.59	87.92	64.99	81.47	77.29	93.06	59.46	87.75	76.81
VIT [4]	61.50	39.61	44.38	39.59	67.46	62.94	89.21	43.14	75.45	69.78
SwinUNet [13]	77.45	27.65	85.47	63.25	80.28	76.45	93.21	56.68	87.63	76.60
R50VIT [4]	71.29	32.87	73.73	55.13	75.80	72.20	91.45	45.98	81.90	73.95
TransUNet [12]	77.48	31.69	87.23	63.13	81.87	77.02	94.08	55.86	85.08	75.62
**MedUNeXt (Ours)**	**79.45**	**23.96**	**89.42**	**65.21**	**82.33**	**78.54**	**94.28**	**60.38**	**88.21**	**77.23**

Bold indicates the best performance in each column. “–” means the value is not reported in the original paper. HD95 is reported in millimeters, DSC in percentages.

**Fig 7:**
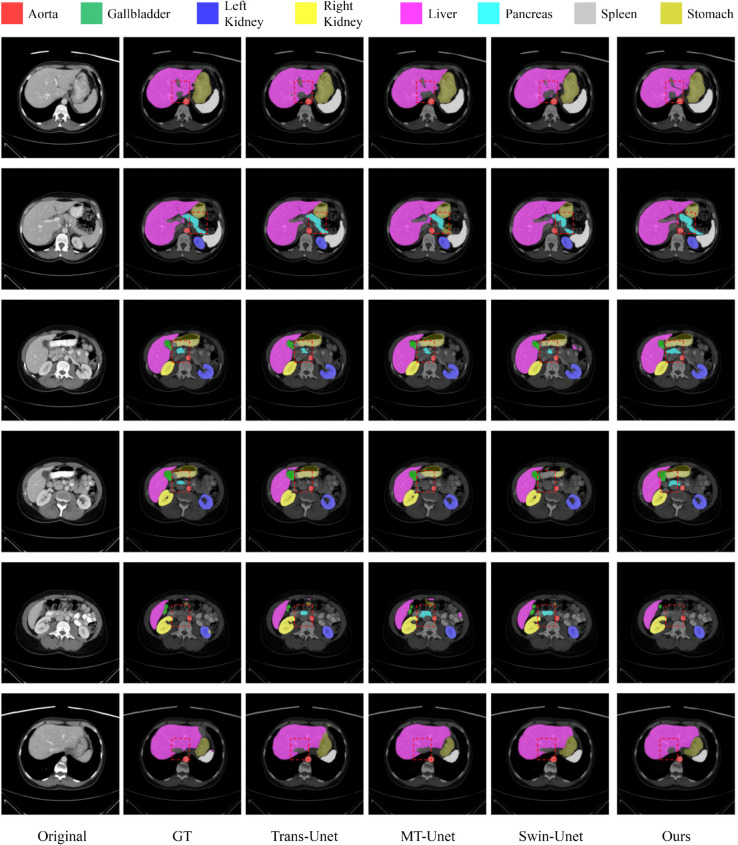
The visualization results of different methods on the Synapse.

In contrast to many Transformer-based approaches, MedUNeXt does not require pretraining, simplifying the training pipeline and reducing computational overhead. Despite relying solely on computationally inexpensive convolution and MLP operations, MedUNeXt achieves state-of-the-art performance when trained from scratch. The visualization results in Fig 7, further demonstrate the segmentation quality of MedUNeXt, which outperforms other methods on the Synapse dataset. These results are particularly relevant in a clinical setting, where accurate segmentation of anatomical structures—such as organs and blood vessels—is critical for tasks such as diagnosis, treatment planning, and surgical intervention. MedUNeXt’s superior performance in segmentation can enhance the precision of organ delineation, thereby supporting applications such as preoperative planning, radiation therapy, and the monitoring of disease progression.

By combining the simplicity of traditional convolutional architectures with the global context awareness of Transformers, MedUNeXt presents a novel perspective for designing visual models in medical image segmentation. Its ability to deliver high segmentation accuracy without the need for pretraining demonstrates its efficiency and scalability, making it a promising approach for a wide range of medical imaging tasks.

### Segmentation task: Research on ACDC

Following the experimental setting on the Synapse dataset, the proposed MedUNeXt was trained from scratch on the ACDC dataset. The experimental results, presented in Table 6, MedUNeXt outperforms both convolution-based methods (e.g., R50-AttnUNet and R50UNet) and Transformer-based methods (e.g., SwinUNet and TransUNet) in terms of Dice Similarity Coefficient (DSC). Specifically, MedUNeXt achieves DSC improvements of 3.32% to 3.79% over convolution-based methods and 1.57% to 3.19% over Transformer-based methods. Notably, MedUNeXt achieves a higher DSC than MT-UNet while utilizing fewer parameters, highlighting its efficiency.

**Table 6:** Segmentation task: Comparison with SOTA methods on the ACDC.

Method	Computational Complexity	Params (M)↓	DSC(%)↑	RV(%)↑	Myo(%)↑	LV(%)↑
UNet [55]	𝒪(N)	41.25	88.09	84.16	86.09	94.01
V-Net [56]	𝒪(N)	46.78	83.47	76.71	83.60	90.12
DARR [57]	$𝒪(N^{2})$	43.56	84.33	77.88	83.86	91.24
R50-ViT [4]	$𝒪(N^{2})$	73.25	87.75	86.35	82.03	94.87
SwinUNet [13]	𝒪(N)	62.20	88.09	86.46	85.79	94.02
R50UNet [12]	𝒪(N)	62.74	87.96	85.15	84.76	93.98
R50AttnUNet [12]	𝒪(N)	57.16	87.49	84.13	84.53	93.80
AttnUNet [42]	𝒪(N)	46.18	88.37	84.12	86.42	94.57
nnUNet [58]	𝒪(N)	40.28	91.22	88.39	89.22	96.05
ViT+CUP [12]	$𝒪(N^{2})$	58.38	84.14	81.22	79.02	92.18
MT-UNet [14]	$𝒪(N^{2})$	75.05	90.43	86.64	89.04	95.62
TransUNet [12]	$𝒪(N^{2})$	105.28	89.71	**88.86**	84.54	95.73
**MedUNeXt (Ours)**	** $𝒪(N\sqrt{N})$ **	**39.85**	**91.28**	**88.10**	**89.42**	**96.31**

Bold indicates the best performance in each column. Computational complexity is noted in Big-O notation, Params in millions (M), and performance is evaluated using Dice (DSC), right ventricle (RV), myocardium (Myo), and left ventricle (LV) segmentation accuracy.

**Fig 8:**
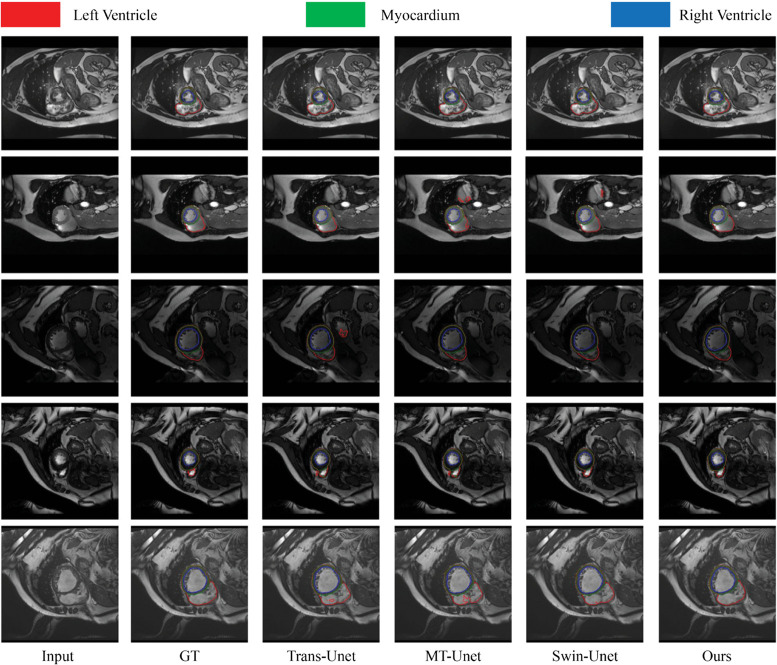
The visualization results of different methods on the ACDC.

MedUNeXt achieves a DSC of 91.28% on the ACDC dataset, outperforming all compared methods while maintaining the lowest computational complexity and parameter count. This outstanding performance reflects MedUNeXt’s ability to accurately delineate complex anatomical boundaries (e.g., left ventricle, myocardium, and right ventricle) even in challenging imaging scenarios such as low contrast or variable morphologies. As shown in Fig 8, MedUNeXt provides precise and consistent segmentation across all three cardiac chambers, surpassing competing models in both boundary clarity and overall region completeness. This level of accuracy is crucial for deriving reliable cardiac metrics, such as ventricular volumes, myocardial thickness, and ejection fraction, which are key to diagnosing and managing cardiovascular diseases.

The visualization in Fig 8 further highlights MedUNeXt’s ability to minimize false positives and false negatives, common issues in medical image segmentation. Compared to other methods, MedUNeXt demonstrates greater consistency across cases and adapts effectively to patient-specific variations and imaging artifacts, ensuring robust and reliable results that support accurate clinical decision-making. In addition to its segmentation accuracy, MedUNeXt’s computational efficiency is evident in its parameter count of 39.85M, significantly lower than TransUNet (105.28M) and MT-UNet (75.05M). This lightweight design enables faster inference and reduced computational costs, making MedUNeXt ideal for real-time applications in resource-constrained settings, such as low-power hospital systems or edge devices. Furthermore, its efficiency ensures compatibility with diverse clinical environments, enabling widespread adoption without compromising performance.

**Segmentation Results Summary.** MedUNeXt achieves state-of-the-art segmentation performance with minimal computational demands, making it a highly efficient and scalable solution for medical image segmentation. Its ability to generalize across diverse patient populations and imaging conditions enhances its clinical utility, offering accurate, robust, and reproducible outcomes in both diagnostic and therapeutic applications. These qualities position MedUNeXt as an excellent tool for real-time cardiovascular imaging, where speed, precision, and reliability are critical.

### Ablation studies

**Fig 9:**
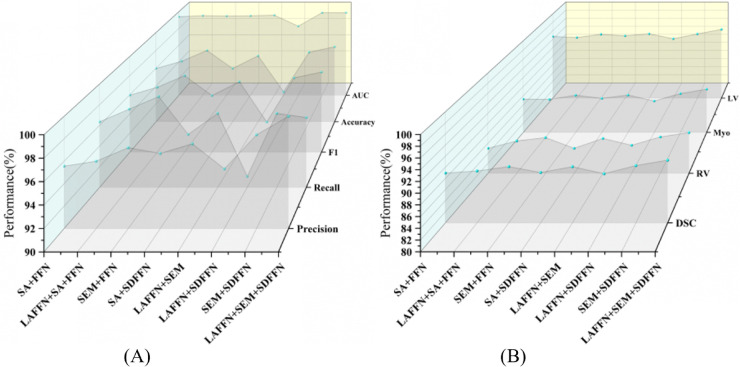
The performance visualization of our variants on two datasets. (A) Ablation study results on SARS-CoV-2 CT-Scan dataset. (B) Ablation study results on ACDC dataset.

To evaluate the contributions of the proposed modules—LAFFN, SEM, and SDFFN—we conducted detailed ablation experiments on the SARS-CoV-2 CT-Scan dataset (for classification) and the ACDC dataset (for segmentation). Specifically, we implement various combinations of our three modules as ablation variants to assess their impact on performance, as listed in Tables 7 and 8, along with the trends visualized in Fig 9. Compared with the the baseline configuration (#1), all three modules show effectiveness in enhancing performance while reducing computational costs. Across both tasks, the proposed modules progressively improve metrics such as F1-score, AUC, and DSC, with the full integration of all modules (#8) achieving the best overall results.

**Table 7:** Ablation study results for classification task.

No.	Method	Params (M)↓	FLOPs (G)↓	Precision↑	Recall↑	F1↑	Accuracy↑	AUC↑
#1	SA+FFN	14.28	2.78	95.56%	96.34%	95.95%	95.97%	0.9939
#2	LAFFN+SA+FFN	16.37	3.09	96.00%	97.56%	96.77%	96.77%	0.9952
#3	SEM+FFN	12.79	2.20	97.20%	98.78%	97.98%	97.98%	0.9947
#4	SA+SDFFN	10.12	2.17	96.69%	95.12%	95.90%	95.96%	0.9950
#5	LAFFN+SEM	9.34	1.69	97.55%	97.35%	97.35%	97.38%	0.9967
#6	LAFFN+SDFFN	5.10	1.07	95.32%	96.09%	93.14%	93.35%	0.9826
#7	SEM+SDFFN	8.63	1.59	98.35%	97.15%	97.75%	97.78%	0.9965
#8	LAFFN+SEM+SDFFN	10.72	1.90	100.00%	96.75%	98.35%	98.39%	0.9988

The baseline configuration is #1 (SA+FFN).

**Table 8:** Ablation study results for segmentation task.

No.	Method	Params (M)↓	FLOPs (G)↓	DSC(%)↑	RV(%)↑	Myo(%)↑	LV(%)↑
#1	SA+FFN	48.25	59.73	88.96%	84.98%	87.26%	94.63%
#2	LAFFN+SA+FFN	59.68	64.69	89.34%	86.44%	87.20%	94.38%
#3	SEM+FFN	43.58	46.36	90.12%	87.11%	88.10%	95.15%
#4	SA+SDFFN	33.09	43.18	89.06%	84.98%	87.42%	94.78%
#5	LAFFN+SEM	22.00	36.59	90.11%	86.94%	88.14%	95.26%
#6	LAFFN+SDFFN	29.28	32.78	88.44%	85.58%	86.64%	94.09%
#7	SEM+SDFFN	24.42	31.94	90.17%	87.34%	87.46%	95.23%
#8	LAFFN+SEM+SDFFN	39.85	39.87	91.28%	88.10%	89.42%	96.31%

The baseline configuration is #1 (SA+FFN).

Benefiting from our ablation variants, we can isolate and evaluate each module’s contribution. For LAFFN, it consistently enhances lesion feature extraction, delivering significant performance gains with minimal computational overhead. For classification, LAFFN (#2 vs. #1 in Table 7) improves the F1-score from 95.95% to 96.77% and the AUC from 0.9939 to 0.9952, with only a slight increase in Params and FLOPs. For segmentation, LAFFN (#2 vs. #1 in Table 8) increases the DSC from 88.96% to 89.34%, with particularly notable improvements in specific regions such as the right ventricle (RV) (84.98% → 86.44%). As illustrated in Fig 9A, LAFFN demonstrates steady improvements across metrics like Precision and Recall, while in Fig 9B, its inclusion significantly enhances segmentation accuracy, particularly for anatomically challenging regions like the myocardium and RV.

SEM plays a pivotal role in achieving a balance between computational efficiency and performance by integrating multi-scale lesion information. For classification, SEM (#3 vs. #1 in Table 7) reduces FLOPs by 20.9% while increasing the F1-score to 97.98%. In segmentation, SEM (#3 vs. #1 in Table 8) improves the DSC to 90.12%, with consistent accuracy gains across regions such as the RV (84.98% → 87.11%) and myocardium (87.26% → 88.10%). As shown in Fig 9B, SEM not only improves overall segmentation accuracy but also enhances the delineation of anatomical boundaries, reinforcing its importance for dense prediction tasks. Its contribution is further underscored by the performance drops observed in configurations lacking SEM (#6/#7), compared to the complete setup (#8).

SDFFN significantly reduces computational costs while maintaining or even improving task performance. For classification, SDFFN (#4 vs. #1 in Table 7) achieves comparable F1-scores while reducing Params by 29.1%. Similarly, in segmentation, SDFFN (#4 vs. #1 in Table 8) reduces Params by 31.4% and FLOPs by 27.7%, with a modest improvement in DSC (88.96% → 89.06%). The efficiency of SDFFN makes it particularly valuable in configurations that combine it with LAFFN and SEM (#8), as reflected in Fig 9B, where its inclusion enhances segmentation consistency and boundary sharpness without incurring significant computational costs.

The full integration of all three modules (#8) achieves the best performance on both tasks, significantly outperforming the baseline. For classification, #8 achieves an F1-score of 98.35%, an AUC of 0.9988, and reduces Params and FLOPs by 25% and 31.7%, respectively, compared to #1. For segmentation, #8 achieves the highest DSC (91.28%), with 17.4% fewer Params and 33.2% fewer FLOPs compared to the baseline. These results are further corroborated by the global trends depicted in Fig 9, where #8 consistently outperforms all other configurations across all metrics. Visualizations reveal LAFFN, SEM, and SDFFN’s complementary roles in enhancing feature representation, multi-scale integration, and computational efficiency.

**Ablation Study Summary.** The ablation experiments validate the lightweight design and high efficacy of the proposed modules. Equipped with them, our method delivers state-of-the-art performance on both lesion classification and organ segmentation tasks, while maintaining computational efficiency. Furthermore, the global trends illustrated in Fig 9 underscore the robustness and efficiency of the MedUNeXt, making it a practical solution for real-world clinical applications.

### Limitations

Despite the strong overall performance and efficiency demonstrated across our experiments, several limitations warrant discussion. First, our current design operates on per-slice 2D inputs and does not explicitly model the inter-slice or temporal dependencies inherent in volumetric CT and cine MRI sequences. Ignoring these dependencies can lead to slice-to-slice discontinuities (*e.g.*, broken vessels, jagged organ boundaries). Second, the method lacks per-instance adaptivity, particularly for small objects. While large-kernel CNNs and MLPs can emulate self-attention and avoid its heavy computational overhead, they inherently constrain per-instance adaptivity. On Synapse in Table 5, although we achieve the best mean DSC and HD95, per-class scores for small organs remain relatively low (Pancreas: 60.38 DSC; Gallbladder: 65.21 DSC).

To address these issues, we will explore adding axial or inter-slice aggregation modules on top of 2D backbones to enforce spatiotemporal consistency while preserving efficiency. In addition, introducing deformable operators or content-conditioned kernels may improve small-object perception without sacrificing overall efficiency.

## Conclusion

In this study, we first identified the key characteristics that visual models should exhibit for effective medical image analysis. Building on these insights, we propose MedNeXt, a novel backbone network for medical image classification that outperforms both CNN-based and Transformer-based architectures on small- and large-scale datasets. We further develop MedUNeXt, a U-shaped segmentation network that integrates multi-scale information within each layer and across the network, achieving state-of-the-art results on general-purpose segmentation benchmarks.

MedNeXt and MedUNeXt strike a better balance between accuracy and computational efficiency across datasets of varying scales, enabling real-time deployment in clinical settings. They are well suited to time-sensitive tasks that demand rapid and precise diagnosis, such as automated screening for monkeypox and other infectious diseases. With computational efficiency and strong generalization, the proposed models can support clinical workflows by enhancing diagnostic performance and reducing the workload on healthcare professionals.

It is important to note that our work primarily focuses on medical image classification and segmentation tasks. Future research will explore the application of our proposed algorithms in tasks such as image alignment, target detection, and super-resolution reconstruction. Overall, our study aims to encourage researchers to develop more effective models and algorithms for medical image analysis by thoughtfully considering specific problems in the field.
